# A Case Report: Psychosis and Phenylketonuria—A Diagnostic Challenge in Adulthood

**DOI:** 10.1192/j.eurpsy.2026.12140

**Published:** 2026-07-31

**Authors:** J. X. Andahua Pinedo, A. Candebat Castellanos, C. C. Pavon Ochoa, M. Gascon Gonzalez, I. Veiga Ramos

**Affiliations:** Psychiatry, University Clinical Hospital of Santiago ~ CHUS, Santiago de Compostela, Spain

## Abstract

**Introduction:**

Phenylketonuria (PKU) is a congenital metabolic disorder caused by a deficiency of phenylalanine hydroxylase (PAH), leading to the accumulation of phenylalanine and subsequent neurotoxicity. It is most commonly detected during infancy through neonatal screening. However, mild or atypical forms may remain undiagnosed until adulthood, presenting with neuropsychiatric manifestations such as cognitive impairment, behavioral disturbances, mood disorders, or psychotic symptoms (Burlina et al., *J Inherit Metab Dis* 2019; 42:584–593). Presentation with overt psychosis is rare and poses a diagnostic challenge in clinical practice.

**Objectives:**

To report a case presenting with a first psychotic episode in the context of a probable late-diagnosed phenylketonuria, and to highlight the importance of considering metabolic causes in the differential diagnosis of atypical neuropsychiatric presentations.

**Methods:**

A 31-year-old male with congenital deafness, delayed language acquisition, and long-standing difficulties in socialization and restricted interests since childhood. No history of substance use, psychiatric follow-up, or prior treatment. On physical examination, obesity and mild dysmorphic features were noted. He was admitted to a psychiatric inpatient unit following an episode of heteroaggressiveness at home, triggered by persecutory delusional ideation centered on his father, accompanied by visual, auditory, and cenesthetic hallucinations with sexual bodily sensations. Cranial computed tomography (CT), magnetic resonance imaging (MRI), and electroencephalogram (EEG) were normal. Basic blood work revealed elevated transaminases and C-reactive protein. Visual evoked potentials showed delayed right prechiasmatic conduction. Given the atypical neurodevelopmental phenotype, a metabolic work-up was requested, including plasma amino acid profile, urinary organic acids, and genetic testing.

**Results:**

Increased plasma phenylalanine levels were found, consistent with possible phenylketonuria. Genetic testing was requested (result pending). During hospitalization, oral antipsychotic treatment with aripiprazole and olanzapine was initiated, resulting in a favorable clinical response. Upon discharge, long-acting injectable aripiprazole was administered, and outpatient follow-up was arranged for definitive metabolic evaluation.

**Conclusions:**

Phenylketonuria may present in adulthood with psychiatric manifestations, including psychosis. Metabolic screening should be included in the assessment of patients with neurodevelopmental disorders, atypical psychosis, or limited response to treatment, as early diagnosis and metabolic control are essential to prevent secondary neuropsychiatric complications.

**Disclosure of Interest:**

None Declared

